# The impact of subacromial impingement syndrome on muscle activity patterns of the shoulder complex: a systematic review of electromyographic studies

**DOI:** 10.1186/1471-2474-11-45

**Published:** 2010-03-09

**Authors:** Rachel Chester, Toby O Smith, Lee Hooper, John Dixon

**Affiliations:** 1 Institute of Health and Social Sciences Research, Faculty of Health, University of East Anglia, Norwich, Norfolk, NR4 7TJ, UK; 2 Physiotherapy Department, Norfolk and Norwich University Hospital, Norwich, NR4 7UY, UK; 3 Institute of Orthopaedics, Norfolk and Norwich University Hospital, Norwich, NR4 7UY, UK; 4 Institute of Biomedical and Clinical Sciences, Faculty of Health, University of East Anglia, Norwich, Norfolk, NR4 7TJ, UK; 5 Health and Social Care Institute, School of Health and Social Care, Teesside University, Middlesbrough, TS1 3BA, UK

## Abstract

**Background:**

Subacromial impingement syndrome (SIS) is a commonly reported cause of shoulder pain. The purpose of this study was to systematically review the literature to examine whether a difference in electromyographic (EMG) activity of the shoulder complex exists between people with SIS and healthy controls.

**Methods:**

Medline, CINAHL, AMED, EMBASE, and grey literature databases were searched from their inception to November 2008. Inclusion, data extraction and trial quality were assessed in duplicate.

**Results:**

Nine studies documented in eleven papers, eight comparing EMG intensity and three comparing EMG onset timing, representing 141 people with SIS and 138 controls were included. Between one and five studies investigated each muscle totalling between 20 and 182 participants. The two highest quality studies of five report a significant increase in EMG intensity in upper trapezius during scaption in subjects with SIS. There was evidence from 2 studies of a delayed activation of lower trapezius in patients with SIS. There was otherwise no evidence of a consistent difference in EMG activity between the shoulders of subjects with painful SIS and healthy controls.

**Conclusions:**

A difference may exist in EMG activity within some muscles, in particular upper and lower trapezius, between people with SIS and healthy controls. These muscles may be targets for clinical interventions aiding rehabilitation for people with SIS. These differences should be investigated in a larger, high quality survey and the effects of therapeutically targeting these muscles in a randomised controlled trial.

## Background

Shoulder pain is a common musculoskeletal problem with a lifetime prevalence of one in three [1]. Up to 54% of sufferers report ongoing pain after 3 years [2]. Subacromial impingement syndrome (SIS), defined as mechanical compression of the rotator cuff and subacromial bursa between the humerus and coraco-acromial arch [3], is the most common cause of shoulder pain, accounting for 40% of shoulder disorders [4]. A recent study in France [5] identified SIS as the most common upper extremity disorder in the working population. This review covers subjects with SIS presenting in the earlier stages of Neer's classification [3] that do not have large or massive rotator cuff tears.

One hypothesised aetiological factor for the development or persistence of SIS has been abnormal muscle activation [6,7]. For example, normal external rotation of the scapula during scaption (shoulder abduction in the plane of the scapula) requires the coordinated action of all parts of trapezius and the serratus anterior, altered synchronisation of which will result in abnormal movement of the scapula and a reduction in upward rotation of the glenoid fossa. Increased downward rotation of the glenoid fossa will reduce the size of the subacromial area and could contribute to the development or persistence of SIS, potentially accounting for the longevity and chronic nature of SIS in the clinical setting.

In order to address kinematic changes which may be contributing to SIS, various therapeutic approaches have been advocated to correct any asynchronous muscle activity, improve dynamic stability and thereby reduce pain and increase function [8,9]. There is however, a lack of cohesive evidence to determine which specific muscles should be targeted during rehabilitation.

Electromyography (EMG) has been used to investigate possible impairments in both the timing and the intensity of muscle activation of the shoulder complex in subjects with SIS compared to subjects with pain free healthy shoulders. With regard to timing, the onset of muscle activation is generally determined as the time point at which the EMG signal from a particular muscle exceeds a set threshold level. The intensity of muscle activation is often denoted by the percentage of maximum voluntary contraction following normalisation of data (%MVC_(EMG)_). There are currently no published systematic reviews which examine and integrate the results of these studies, some of which report contradictory findings. The purpose of this study was therefore to systematically review the literature to examine whether a difference exists in activation of the shoulder complex of people with SIS compared to healthy controls.

## Methods

### Eligibility Criteria

We included published and unpublished primary studies in English, comparing EMG activity of the shoulder complex in adults (aged 16 years or more) with painful SIS (including additional rotator cuff tendinopathy or tendinosis) and adults without shoulder pain. Pathologies such as glenohumeral instability, large or massive rotator cuff tears, rotator cuff pathology in the absence of a description of subacromial impingement syndrome and non-specific shoulder pain were excluded. Animal and cadaver studies and single-subject case reports were also excluded.

### Search Strategy

We searched Medline, CINAHL, AMED and EMBASE via Ovid from inception to November 2008 using indexing, text terms and Boolean operators. The full Medline search strategy is presented in additional file 1. We also searched SIGLE (System for Information on Grey Literature in Europe), National Technical Information Service, National Research Register (UK), the British Library's Integrated Catalogue, and Current Controlled Trials to November 2008 for unpublished or grey literature. Reference lists of review papers and all papers assessed in full text for inclusion were checked for further studies.

### Study Selection

Two investigators (RC, TS) independently evaluated all identified titles and abstracts against the pre-defined eligibility criteria. Full manuscripts of those which potentially adhered to the eligibility criteria were ordered and screened independently for inclusion (RC, TS). When disagreement arose in study eligibility, and a consensus could not be reached the plan was that any disagreement would be settled by an adjudicator (JD). The adjudicator was however not required.

### Data Extraction

Data from each included study was entered onto a data extraction form by a single investigator (RC). Each form was re-evaluated and verified by a second investigator (TS) tabulating: author names and publication date; study design; sample size; population characteristics including diagnosis, subject age and gender, history of pathology; method of diagnosis; method of electomyographic data collection; glenohumeral activities under assessment; statistical analysis; results; and any relevant methodological limitations. Method of electromyographic data collection, including reproducibility of electrode positioning, reliability of EMG equipment, and extracted results were further re-evaluated and verified by a third investigator (JD) with particular expertise in EMG analysis.

Where necessary, standard deviations (sd) of percentage of maximum voluntary contraction (%MVC_(EMG)_) and EMG onset timing data were calculated from standard errors or confidence intervals. The corresponding author of any studies which did not provide sufficient data in the text to determine the mean and sd were contacted by email to request further details. If no response was received, contact details of additional authors were sought on the internet and contacted by email. If no response was received, where possible, we acquired data based on graphical illustration in published papers. If disagreement arose and a consensus could not be reached, the plan was that any disagreement would be settled by the third investigator or adjudicator (JD). No disagreements arose which could not be resolved by discussion and always involved clarity of information, sometimes involving the third investigator.

### Assessment of Trial Quality

All included studies were assessed for methodological quality independently in duplicate (RC, TS). Criteria for assessment were based on the Critical Appraisal Skills Programme (CASP) tool for observational studies recommended by the Public Health Resources Unit of the NHS [10]. At present, to the authors' knowledge there is no validated assessment or scoring system for observational studies appropriate to our research question. Our scoring tool and selection of criteria presented in table one was therefore adapted from the CASP tool by the authors to provide a framework for presenting the most pertinent methodological issues for our research question. Based on 6 key criteria (matching of subjects and controls, power calculation justifying sample size, reproducibility of electrode position - this could include a reference for further details, reliability of EMG equipment, tester blind to group allocation, and sufficient results in the text or supplied by the authors) a maximum score of 6 was awarded to each study. When information about any of the 6 key criteria was not provided in the paper, a score of zero was given for that particular criteria. A third investigator (JD) verified the results. If disagreement arose and a consensus could not be reached, the plan was that any disagreement would be settled by the third investigator who could, if necessary, act as an adjudicator (JD). No disagreements arose which could not be resolved with discussion and always involved clarity of information within the text, on one occasion involving all the authors (see foot note table 1).

**Table 1:** Study design and methodological issues

Study/country of origin	Subj. selection	Controlmatch	Justification. for sample size:	Electrode Position Reproducible	Reliability tested	Tester blind to group allocation	Sufficient results in text or supplied by authors	Score
Score		1	1	1	1	1	1	
Bandholm 2006 Denmark [6]	NS	Y	NS	Y	NS	NS	**Y	3
Brox 1997 Norway [12]	N	NS	NS	Y	N	NS	N~	1
Clisby 2008 Australia [13]	NS	N	NS	Y	NS	NS	N~	1
Cools 2003 [18] 2007 [14] Belg.	NS	Y	Y	Y	Y N	NS	Y Y	5 4
Finley 2005 USA [16]	NS	Y	NS	Y	NS	NS	**Y	3
Ludewig 2000 USA [7]	Conv	Y	Y	Y	Y	NS	**Y	5
2008 Moraes [19] DeMorais-Faria [15] Brazil	NS	Y	NS Y	***Y	NS	NS	N Y	2 4
Reddy 2000 USA [17]	NS	NS	NS	NS	NS	NS	N	0
Wadsworth 1997 USA [20]	Conv	*Y/N	N	***Y	Y/N*	N	N~	2

Abbreviations: Com.obs - Comparative observational,. Conv - convenience, NS - not stated, NA - Not applicable, Y - Yes, N - No. Subj. - subject, *Incomplete data, ** Authors provided on request,. *** Via reference, ~in chart form

Note: The reviewers recorded that matching of subjects and controls took place when this was stated by the authors and accompanied by supporting demographic details in the text. When full demographic details (mean age, SD and population for selection) were provided in the text without an indication of whether or not there was active matching the reviewers made their own judgement based on predetermined criteria; matching was judged to have taken place when all participants in both groups were selected from similar sporting or occupational activities and when the mean age and SD place did not differ more than 5 years.

### Analysis

We intended to perform meta-analyses to assess mean differences between SIS and healthy control EMG activity. However there was substantial heterogeneity between studies with regards to methods of assessment, functional tasks, and muscles evaluated. Accordingly, meta-analyses were inappropriate. We have tabulated data following a narrative review format, noting significant differences in EMG activity of the shoulder complex.

The combination of muscles and movements assessed varied by study, and few studies investigated the same muscles for the same task. The results have been presented and summarised for each individual muscle. Each muscle was assessed by EMG intensity (%MVC_(EMG)_) and by the EMG timing if available. A p value of 0.05 is taken as the level of statistical significance. Exact values are provided in the results section as stated in the text of each paper unless otherwise indicated. Results presented in any tables, figures or additional files are the reviewers' calculations based on the mean and sd of each group provided within each study paper, communicating authors, or calculated/estimated from charts.

## Results

### Study Characteristics

**Figure 1:**
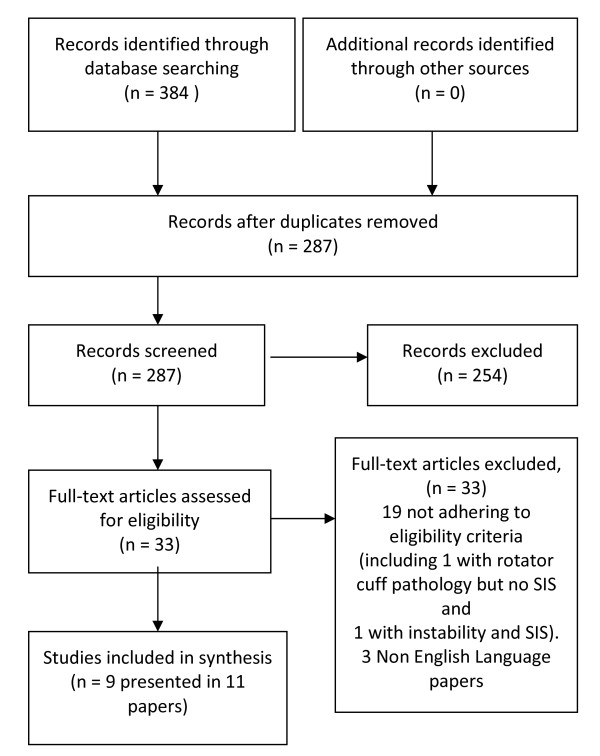
**PRISMA Flow Diagram mapping the review**. *From *Moher D, Liberati A, Tetzlaff J, Altman DG, The PRISMA Group (2009). *P*referred *R*eporting *I*tems for *S*ystematic Reviews and *M*eta-*A*nalyses: The PRISMA Statement. PLoS Med 6(6): e1000097 [11]

The study flow is demonstrated in Figure 1[11]. Of 384 citations identified, eleven, describing nine studies, met the inclusion criteria. All studies were comparative observational/case-control designs. Eight studies compared mean differences in normalised EMG intensity (%MVC _EMG_) during active and functional tasks [6,7,12-17], and three studies compared EMG onset timing [18-20], two studies during active movement [19,20] and one study during the reaction when the arm was unexpectedly released from a passive support [18]. Study characteristics are presented in additional file 2.

In total, 141 subjects with SIS and 138 healthy control subjects were included in the review. Sample sizes ranged from 18 to 69 subjects. The youngest participant was 16 years, the oldest 66 years. Two studies stated that a convenience sampling strategy was used to recruit subjects and controls [7,20], for other studies participant selection methods were unclear. Studies were carried out in the USA [7,16,17,20], South America [15,19], Australia [13] and Northern Europe [6,12,14,18]. SIS duration ranged from three weeks [20] to 10 years [7] and was unclear in 2 studies [13,16]. Only two studies indicated the presence or absence of shoulder pain during testing, both of which provided an indication of the pain intensity their symptomatic subjects reported [6,12].

Six studies used surface EMG [7,13-16,18], one used intramuscular EMG [17] and two used both [6,12] depending on the muscle investigated. Eight studies provided complete results (mean and sd) for subjects with and without SIS for at least some outcomes in the text [12,14,15], by correspondence [6,7,16] or measured from figures [12,13,20]. In one study [12], EMG results were partly normalised by the reviewers, allowing comparison of mean %MVC_EMG _and sds between studies.

Assessment of trial quality is presented in Table 1. The potential for examiner bias did not appear to be controlled in any included study; no studies indicated whether the researcher was blinded to group allocation. Subjects and controls were matched in five studies [6,7,14-16,18,19]. Electrode position was reproducible in eight studies [6,7,12-16,18-20]. The number of tests prior to data collection, and whether results were recorded as a single test or mean of several, varied. Three studies indicated that reliability of EMG equipment was assessed [7,18,20]. Ludewig and Cook [7] reported all ICCs for within-day trial to trail reliability to be between 0.73 and 0.89 for all their EMG amplitude data. Cools et al [18] similarly present test retest ICC values of between 0.71 and 0.78 for of muscle latency. Both of these indicate good reliability. Wadsworth and Bullock-Saxton [20] examine the variability of EMG onset timing between shoulders with SIS and control shoulders over an unspecified period of time. This study reported a statistically significant difference in results implying that subjects with SIS display greater intra-subject variability on the affected side, using a one sided F test. This is difficult to interpret and not a usual method to assess reliability. Three studies provided justification for the selected sample size [7,14,15,18,19]. Three studies (covered in four papers) scored ≥ 4 [7,14,15,18] on our scoring criteria.

### Comparison of %MVC_(EMG) _for each muscle

Three studies (n = 67) compared mean differences in EMG activity for the Supraspinatus [6,12,17]. See additional file 3. All studies used intramuscular electromyography. Using hand weights or isokinetic apparatus to produce torques between 20% and 35% MVC, procedures included concentric and eccentric scaption and isometric abduction at 45 and 90 degrees. None of the studies demonstrated a significant difference in EMG activity between groups.

These same three studies and Clisby et al [13] compared mean differences in EMG activity for infraspinatus and middle deltoid using a combination of surface and intramuscular electromyography (n = 99). See additional file 4. In addition to the procedures outlined previously Clisby [13] investigated isometric external rotation. Reddy et al [17] reported significantly decreased EMG in infraspinatus activity during 30-90° concentric scaption and middle deltoid during 60-90° concentric scaption in subjects with SIS in comparison with controls (p < 0.05). Within the paper only mean differences were presented with no details of variation from the mean or exact significance. No additional significant differences were demonstrated between subjects with and without SIS.

Of the three studies investigating isometric activity, Clisby et al [15] reported a reduction in EMG activity within middle deltoid in subjects with SIS in comparison to those without during external rotation at 70% MVC (*p *= 0.042) but not at 10% or 40% MVC. In contrast, although not reported, the authors of this review noted an increase in EMG activity in middle deltoid in subjects with SIS in Brox et al's [12] study during abduction at a torque of 25%MVC. This difference did not remain at exhaustion and was not present upon re-testing after 10 minutes recovery time.

One study [17] (n = 30) compared mean differences in EMG activity for the subscapularis and teres minor using intramuscular electromyography. The paper reports a significant decrease in activity of subscapularis between 30-60° of scaption in subjects with SIS compared to healthy controls (p < 0.05), but no additional significant differences between the groups (p > 0.05). Within this paper only mean differences are presented with no details of variation from the mean and exact significance.

Finley [16] (n = 23) compared mean differences in EMG activity for Biceps, using surface electromyography and reported no significant difference during transfers towards or away from the painful shoulder between a cohort of wheelchair users with and without SIS.

Four studies (n = 113) compared mean differences in EMG activity for the Serratus Anterior [6,7,15,16] using surface electromyography. Procedures included concentric and eccentric scaption and isometric abduction at 90 degrees without an external load or using hand weights or isokinetic apparatus to produce torques between 20% and 35% MVC. In addition one study investigated wheelchair transfers in a cohort of wheelchair users [16]. Three of the four studies reported that there was no significant difference between groups (p < 0.05) [6,15,16]. Ludewig et al [7], the highest scoring study based on our 6 criteria, extended their analysis and report "a main effect for group" (p < 0.05). Our observation of the raw data (see table 2) indicates a trend towards a decrease in EMG activity in subjects with SIS in comparison with controls during concentric and eccentric scaption, but not during wheelchair transfers.

**Table 2:** Mean differences (Mean diff.) 95% confidence intervals (95%CI) and statistical significance of differences in Serratus Anterior %MVC_(EMG) _activity between subjects with (Subjects) and without (Controls) SIS.

Author	Task	Torque as %MVC_(EMG)_	Subjects	Controls	**Mean Diff**.	95%CI	**Stat. sig**.
	Concentric Scaption						
Ludewig	< 60°	No load	8.6 ± 6.9	13.5 ± 8.7	-4.90	-9.3, -0.6	*0.03
Ludewig		2.3 kg	17.2 ± 9.5	25.9 ± 13.1	-8.7	-15.0, -2.4	*0.007
Ludewig		4.6 kg	30.2 ± 17.6	46.3 ± 23.6	-16.10	-27.6, -4.6	*0.006
Bandholm		20% MVC	18.86 ± 4.32	22.72 ± 10.43	-3.86	-11.24, 3.52	0.31
Bandholm		27.5% MVC	22.76 ± 5.3	28.47 ± 10.51	-5.71	-13.4, 1.98	0.15
Bandholm		35% MVC	30.28 ± 6.4	35.87 ± 13.73	-5.59	-15.49, 4.31	0.27
							
Ludewig	61-90°	No load	14.7 ± 7.7	20.9 ± 10.3	-6.20	-11.24, -1.16	*0.02
Ludewig		2.3 kg	29.4 ± 11.2	38.7 ± 18.6	-9.30	-17.81, -0.79	*0.03
Ludewig		4.6 kg	53 ± 19.5	64.9 ± 29	-11.90	-25.6, 1.8	0.09

Ludewig	> 90°	No load	28.1 17.1	33.1 16.9	-5.00	-14.4, 4.42	0.30
Ludewig		2.3 kg	45.1 17.2	55.4 22.9	-10.30	-21.5, 0.9	0.07
Ludewig		4.6 kg	79.2 28.5	89.0 36.5	-9.8	-28.0, 8.4	0.29
Bandholm		20% MVC	34.7 8.2	36.9 14.7	-2.23	-15.6, 9.1	0.70
Bandholm		27.5% MVC	45.9 15.2	47.5 21.3	-1.58	-18.7, 15.5	0.86
Bandholm		35% MVC	55.6 16.2	61.8 22.3	-6.2	-24.1, 11.9	0.50
							
	Eccentric Scaption						
Bandholm	< 60°	20% MVC	11.7 3.0	14.9 9.0	-3.27	-9.5, 2.9	0.30
Bandholm		27.5% MVC	17.5 4.0	21.5 7.9	-4.00	-9.8, 1.8	0.17
Bandholm		35% MVC	21.3 6.7	28.7 9.0	-7.41	-14.6, -0.1	0.05

Bandholm	> 90°	20% MVC	28.9 7.75	29.0 11.0	-0.12	-8.9, 8.7	0.98
Bandholm		27.5% MVC	37.8 9.7	39.2 16.6	-1.41	-14,0, 11.2	0.83
Bandholm		35% MVC	44.6 11.2	52.7 19.7	-8.07	-22.9, 6.7	0.29

DeMorais Faria	160-0°	No load	21.3 12.6	28.6 8.3	-7.30	-16.7, 2.05	0.13
							
	Wheelchair transfers						
Findley	0-30	Towards	43.8 63.5	44.8 21.5	-1.00	-46.8, 44.8	0.97
	31-60	unaffected	32.1 34.2	47.8 50	-15.70	-49.5, 18.1	0.36
	> 61	limb	43.8 38.9	36.2 47	-1.40	-52.8, 50.0	0.96
							
							
	0-30	Towards	40 30.8	22.4 21	17.6	-10.3, 45.5	0.22
	31-60	affected	27.5 40.9	23.1 28.1	4.40	-24.7, 33.5	0.77
	> 61	limb	9.6 11.7	13 10.1	-3.40	-14.2, 7.4	0.54

These same four studies [6,7,15,16] and Cools et al [14] (n = 180) compared mean differences in EMG activity for the Upper Trapezius, all using surface electromyography. In addition to the procedures outlined previously, Cools investigated isokinetic concentric abduction and external rotation [14]. The results are displayed in table 3. Increased EMG activity in subjects with SIS compared to subjects in the control group were observed during loaded and unloaded scaption and were reported as statistically significant for scaption greater than 90° whilst carrying a 4.6 kg load (p < 0.05) [7] and during isokinetic abduction (p < 0.001) and external rotation (p < 0.001) [14]. These studies scored the highest at 4 and above on the reviewers scoring criteria. The direction and significance of these differences was not consistent across other studies.

**Table 3:** Mean differences (Mean diff.) 95% confidence intervals (95%CI) and statistical significance of differences in Upper Trapezius %MVC_(EMG) _activity between subjects with (Subjects) and without (Controls) SIS.

Author	Task	Torque as %MVC_(EMG)_	Subjects	Controls	**Mean Diff**.	95%CI	**Stat. sig**.
	Concentric Scaption						
Ludewig	< 60°	No load	22.5 ± 13.1	17.1 ± 10.9	5.40	-1.3, 12.1	0.11
Ludewig		2.3 kg	36.9 ± 18.7	31.1 ± 15.5	5.80	-3.7, 15.3	0.23
Ludewig		4.6 kg	56 2 ± 4.9	53.7 ± 22.6	2.30	-10.8, 15.5	0.73
Bandholm		20% MVC	13.8 ± 2.7	17.2 ± 6.3	-3.27	-7.8, 1.21	0.15
Bandholm		27.5% MVC	16.7 4 ± 8	20.4 ± 6.25	-3.70	-8.9, 1.45	0.16
Bandholm		35% MVC	21.8 ± 6.32	24.3 ± 7.8	-2.50	-9.1, 4.1	0.46

Ludewig	61-90°	No load	29.0 ± 13.7	22.6 ± 10.9	6.40	-0.5, 13.3	0.07
Ludewig		2.3 kg	48.4 ± 21.5	39.5 ± 14.2	8.90	-1.2, 19.0	0.08
Ludewig		4.6 kg	76.5 ± 26.0	65.2 ± 21.8	11.30	-2.0, 24.6	0.10

Ludewig	> 90°	No load	31.5 ± 13.9	25.5 ± 10.9	6.00	-0.9, 12.9	0.09
Ludewig		2.3 kg	47.4 ± 20.3	42.6 ± 14.6	4.80	-5.0, 14.6	0.34
Ludewig		4.6 kg	77.3 ± 22.5	66.8 ± 20.0	10.5	-1.3, 22.3	0.08
Bandholm		20% MVC	24.3 ± 8.13	24.4 ± 8.6	-0.04	-7.6, 7.7	0.99
Bandholm		27.5% MVC	31.0 ± 10.5	33.4 ± 12.4	-2.43	-13.0, 8.2	0.65
Bandholm		35% MVC	42.6 ± 14.5	46.6 ± 16.7	-3.97	-18.5, 10.4	0.59
							
Cools	Isokinetic abduction		94.7 ± 27	73 1 ± 9.4	-21.72	-32.7, -10.8	*<0.001
							
Cools	Isokinetic external		70.5 ± 18.9	45.2 ± 12.2	-25.30	-17.9, 32.7	*<0.001
	Rot'n						

	Eccentric Scaption						
Bandholm	< 60°	20% MVC	15.6 ± 4.9	15.0 ± 5.9	0.59	-4.4, 5.6	0.82
Bandholm		27.5% MVC	20.2 ± 5.8	21.2 ± 5.6	-0.99	-6.3, 4.3	0.71
Bandholm		35% MVC	24.3 ± 6.3	28.0 ± 6.3	-3.78	-9.6, 2.1	0.20
							
Bandholm	> 90°	20% MVC	19.9 ± 6.6	19.6 ± 8.3	0.32	-6.6, 7.2	0.93
Bandholm		27.5% MVC	25.9 ± 6.7	28.2 ± 9.8	-2.3	-10.0, 5.4	0.56
Bandholm		35% MVC	36.8 ± 11.0	41.2 ± 12.0	-4.39	-15.0, 6.2	0.42

Morais Faria	160-0°	No load	16.8 ± 9.3	18.4 ± 8.5	-1.60	-9.4, 6.2	0.69
							
	Wheelchair transfers						
Findley	0-30	Towards	5.8 ± 4.6	9.2 ± 7.5	-3.40	-8.9, 2.0	0.22
	31-60	unaffected	6.7 ± 11.5	7.3 ± 7.5	-0.60	-9.0, 7.8	0.89
	> 61	limb	15.3 ± 22.4	7.8 ± 5.0	7.50	-18.0, 33.0	0.56
							
	0-30	Towards	8.0 ± 9.9	5.5 ± 4.1	2.50	-5.8, 10.8	0.56
	31-60	affected	9.5 ± 8.5	9.9 ± 18.4	-0.40	-11.1, 10.3	0.94
	> 61	limb	4.3 ± 2.4	8.6 ± 5.7	-4.30	-7.7, -0.9	*0.01

Three studies (n = 108) compared mean differences in EMG activity for the Middle Trapezius [14,15,17] all using surface electromyography. See additional file 5. Procedures included isokinetic concentric abduction and external rotation, eccentric scaption and isometric abduction at 45°. One of the two higher scoring studies demonstrated significantly lower EMG activity during isokinetic external rotation in subjects with SIS compared to those in a control group (p < 0.01) [14]. The direction and significance of any differences was not consistent across other procedures.

Five studies (n = 182) compared mean differences in EMG activity for the Lower Trapezius [6,7,14-16]. All used surface electromyography. See additional file 6. Procedures involved isokinetic concentric abduction and external rotation, concentric and eccentric scaption with and without loads, isometric abduction at 90° and wheelchair transfers. Three of the five studies demonstrated no significant differences between groups [6,15,16]. Two of three studies scoring 4 or above, provided significant but conflicting results. Cools et al [14] reported significantly lower EMG activity in subjects with SIS (p = 0.003) during isokinetic abduction whilst Ludewig and Cook [7] report a significantly greater EMG activity for subjects with SIS between 61° to 90° and >90° concentric scaption during all loads.

Two studies (n = 41) compared mean differences in EMG activity in anterior deltoid [6,16] and one (n = 32) in posterior deltoid [13]. No significant differences between groups were demonstrated during activities which included concentric and eccentric scaption, isometric external rotation and wheelchair transfers.

Bandholm [6] (n = 20) compared the mean difference in EMG activity for Latissimus Dorsi using surface electromyography during concentric and eccentric scaption and isometric abduction. A significant increase in activity was demonstrated at 20% MVC between 45° to 60° concentric abduction in subjects with SIS in comparison with controls (p = 0.05) but not at higher MVCs or through other ranges of movement.

No study compared the mean difference in EMG activity for the Rhomboids and Pectoralis Major.

### Onset of Muscle Activation

**Figure 2:**
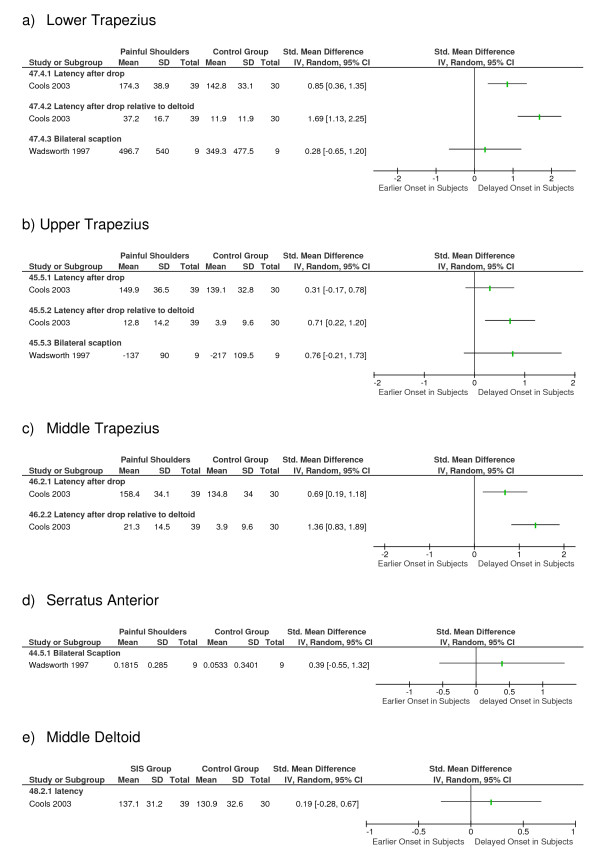
**Mean differences and 95% Confidence Intervals (95%CI) for differences in EMG onset times between subjects with (Subjects) and without (Controls) SIS**.

Three studies [18-20] (n = 107) investigated differences in muscle activation times between participants with and without SIS using surface EMG. Two studies [19,20] evaluated onset times on initiating bilateral scaption in standing (n = 38) and one study [18] evaluated onset during a reaction when the arm was suddenly and unexpectedly released from a passive support (n = 69). See figure 2. Both Wadsworth [20] and Moraes [19] reported a greater variability, the former significantly so, in muscle activation times for subjects with painful shoulders in comparison with subjects with healthy shoulders.

All three studies (n = 107) investigated the lower trapezius. Cools [18] and Wadsworth [20] demonstrated a significant delay (p < 0.01) in EMG onset in the affected shoulder of subjects with SIS in comparison to the shoulders of subjects in the control group. Moraes [19] did not detect differences between groups.

Two studies (n = 89) investigated timing in the middle trapezius. Cools et al [18] demonstrated a delayed activation (p < 0.01) in the affected shoulder in SIS subjects compared to the dominant shoulder of control subjects. Moraes [19] did not detect any significant differences between groups. Cools [18] also reported that the onset of both lower and middle trapezius relative to that of the middle deltoid was delayed in SIS.

All three studies (n = 107) [18-20] compared onset times of the upper trapezius, and found no significant differences between groups.

Two studies (n = 38) compared onset times of the serratus anterior, both during the commencement of scaption [19,20] and demonstrated no difference between the painful shoulder of subjects with SIS and a control group of healthy shoulders.

One study (n = 69) investigated onset times of middle deltoid during a reaction response [18] and reported no difference between the groups.

## Discussion

### Results

This systematic review does not provide evidence to suggest a significant difference in %MVC_EMG _activity between the shoulders of subjects with painful SIS and the healthy controls in the following muscles: supraspinatus, teres minor, biceps, serratus anterior, anterior and posterior deltoid. There is limited or conflicting evidence to suggest that a difference in %MVC_EMG _may be demonstrable during some tasks for the following muscles: infraspinatus, subscapularis, upper, middle and lower trapezius, middle deltoid and latissimus dorsi activity. Increased %MVC_EMG _in the upper trapezius during scaption was the most consistent finding in two higher quality studies. This was not supported in three smaller, lower quality studies. Changes in recruitment patterns were more consistent across reported results for two of three studies. There was a consistent delay in the onset of lower trapezius during scaption, and in the reaction test when the arm was suddenly and unexpectedly released from a passive support. There was also a delay in onset of upper trapezius and serratus anterior compared to deltoid during the reaction test.

### Statistical Considerations

Although trends sometimes appeared to exist between subjects with and without SIS, there were limited statistically significant differences. The three largest studies reviewed most commonly reported significant differences between groups [7,14,18] and were the only three that stated the power of their study. A number of studies may have suffered from a type II error in which there were differences between groups but the study was not adequately powered to detect these. The between-subject variability in EMG values, particularly subjects with SIS [19,20], may require larger sample sizes to provide adequate power to detect true differences. It remains unclear whether more significant differences in EMG activity do exist between the two groups and we recommend that sample sizes for future studies be based on a power calculation.

### Interpretation of EMG Analysis

There are a number of methodological factors in EMG that should be borne in mind when trying to interpret the findings of this review. The shoulder complex is a particularly difficult area to study with EMG, and researchers should be applauded for attempting to shed light on this topic. The issue of crosstalk, the detection of EMG signals from muscles other than the one of interest, may be a problem in the shoulder musculature when using surface EMG on relatively small muscles. Methodologies differed markedly between studies, which made comparison difficult.

There are major points to consider regarding the interpretation of EMG intensity. Normalisation contractions, usually maximal, must be carried out to allow comparisons between groups [21]. For normalisation contractions, some studies have carried out *one *reference task maximally for the *all *muscles investigated, rather than carrying out separate contractions for each muscle which is probably impractical. However this may mean that activation is not maximal in all muscles during this one task. Also, importantly, in people with painful conditions the interpretation of EMG data normalised to %MVC_(EMG) _needs careful consideration. If participants with SIS cannot or do not fully activate their muscles during the normalisation contraction, whether because of pain, inhibitory mechanisms, or avoidance, then %MVC_(EMG) _values may be affected [12,21] as the 100% levels are not true maximal values. If this only occurs in the SIS group and not the control group (or contralateral limb) as is probable, then it will be a possible confounding factor, inflating the SIS normalised EMG levels during functional activity, when the true effect is on the normalisation contraction. The paper by Brox [12] used this very point as a rationale for also analysing non-normalised data. This issue is often not discussed in the literature, but may be one possible reason for differences between studies included here, for example the findings of reduced lower trapezius activation by Cools et al [14], compared to the higher activation reported by Ludewig and Cook [7]. We recommend that researchers carefully consider all of the possible issues with normalisation in designing future studies.

Similarly, regarding EMG timing to assess the temporal aspects of muscle recruitment, different methods of determining EMG onset are available, and were in fact used in the included studies. Wadsworth and Bullock-Saxton [20] determined onset as the point at which the EMG signal exceeded 5% of its maximum amplitude, whereas Cools et al [18] used 10% of maximum as the threshold value. Here the onset point is clearly relative to the maximum signal amplitude. In contrast, Moraes et al [19] identified onset as the point at which the EMG signal exceeded the resting baseline level by over 2 sds, so that onset is relative to baseline levels. Therefore it is possible that the differences between study findings on muscle recruitment timing, with some delayed activation observed by Cools et al [18], and Wadsworth and Bullock-Saxton [20], but not by Moraes et al [19], could be due to methodological differences.

Only three [17,18,20] of the included studies assessed reliability. Regarding reliability it is important to be aware that much depends on the equipment and techniques used (e.g. indwelling or surface electrodes, surface electrode size), the experience of the data collector, and the outcome measures (e.g. timing or intensity) and how they are determined. Because methodologies differ between studies, findings from studies that have not assessed the reliability of their specific protocol and outcome measures should be interpreted with caution.

### Demographic and Procedural Considerations

The interpretation of results may be influenced by a number of methodological issues. Only two studies indicated the presence or absence of shoulder pain during testing [6,12]. Changes in shoulder pain, produced by a local injection of subacromial anaesthetic, have been demonstrated to cause immediate change in EMG activity in some shoulder muscles [22]. The demographic characteristics of participants within the studies varied and this may have contributed to the heterogeneous results between studies. Occupational, recreational, and ambulatory demands upon the affected shoulder varied considerably, as did the duration of symptoms and whether subjects had received previous physiotherapy. Matching of subjects and controls was not documented or did not take place [12,13,17] or details incomplete [20] in four studies and may have accounted for differences between groups. The potential for examiner bias did not appear to be controlled in any included study. It was not possible to ascertain the impact of these specific factors on our results, particularly given the procedural heterogeneity between studies.

Procedural details may have contributed to the heterogeneous results. Isokinetic apparatus was used in four studies and can provide a reproducible and standardised method of assessment [23] but is less representative of the participants function than free active movement without trunk restraint, the later in turn more difficult to control. Five studies investigated isotonic scaption, all of which varied in terms of standing/sitting position, apparatus, speed, or load. As a result, the differences seen in results for a similar direction of movement may be attributed to the varying type of muscle action required, as opposed to the actual discrepancies in results across studies.

### Consideration of Scoring Criteria

The absence of a validated assessment or scoring system for observational studies appropriate to our research question required that we develop our own criteria based on CASP [10]. Whilst agreement between authors was high in this study, our scoring system has not been tested for reliability. The primary objective of our selected criteria and scoring system was to facilitate the clear presentation of our quality assessment. Results should be interpreted within this context.

### Considerations for Clinical Practice and Future Research

As stated previously normal external rotation of the scapular and upward rotation of the glenoid fossa to avoid potential SIS during scaption requires the coordinated action of all parts of trapezius and the serratus anterior. A longitudinal study is recommended to determine whether electromyography findings change as SIS develops and thereby guide the selection of rehabilitation techniques, if appropriate, upon the longevity of symptoms.

Nociceptive input may influence peripheral and central motor control [24]. Two of three studies investigating the timing or onset of muscle recruitment in this review demonstrated changes in the unaffected as well as the painful shoulder [18,20], indicating the possibility of a more global response. These results have implications for the selection of appropriate rehabilitation techniques and may be an aetiological factor for the incidence of bilateral SIS, rather than a local mechanical insult.

If selected muscles, such as upper and lower trapezius, are important to preferentially rehabilitate, future studies are recommended to examine whether these findings represent the whole population of patients with SIS, or whether this is typical of specific sub-groups. These studies should control for examiner bias, state the presence, or absence of shoulder pain during testing, and demonstrate the reliability of their procedure. Well designed clinical trials are then recommended to determine the efficacy of specific rehabilitation programmes targeting these muscles in clearly defined populations.

## Conclusion

A difference may exist in %MVC_(EMG) _for upper trapezius and onset of muscle recruitment for lower trapezius between people with SIS and healthy controls. Evidence did not exist or was inconsistent to support differences in other muscles. The variation in results may be typical of the range of patients presenting with SIS or may be because some studies were inadequately powered to detect true differences. Methodological and demographic heterogeneity may also be a source of variation between studies.

## Competing interests

The authors declare that they have no competing interests.

## Authors' contributions

RC co-coordinated the review, and contributed to the literature search, data extraction, data analysis and drafting of the manuscript. TS contributed to the literature search, data extraction, data analysis and drafting of the manuscript. LH provided expert advice on systematic reviewing and contributed to data analysis and drafting of the manuscript. JD provided expertise on EMG analysis and contributed to data extraction, data analysis and drafting of the manuscript. All authors read and approved the final manuscript.

## Pre-publication history

The pre-publication history for this paper can be accessed here:

http://www.biomedcentral.com/1471-2474/11/45/prepub

## Supplementary Material

Additional file 1 **Search strategy**. Description of MESH terms and text words used in the literature search Click here for file

Additional file 2 **Population characteristics and procedural details**. Subject and control characteristics, details of task and EMG collection Click here for file

Additional file 3 Mean differences (Mean diff.) 95% confidence intervals (95%CI) and statistical significance of differences in Supraspinatus %MVC_(EMG) _activity between subjects with (Subjects) and without (Controls) SIS. Click here for file

Additional file 4 Mean differences (Mean diff.) 95% confidence intervals (95%CI) and statistical significance of differences in Infraspinatus %MVC_(EMG) _activity between subjects with (Subjects) and without (Controls) SIS. Click here for file

Additional file 5 Mean differences and 95% confidence intervals (95%CI) for differences in Middle Trapezius %MVC_(EMG) _activity between subjects with (Subjects) and without (Controls) SIS. Click here for file

Additional file 6 Mean differences and 95% Confidence Intervals of differences in Lower Trapezius EMG activity between subjects with (Subjects) and without (Controls) SIS. Click here for file
