# Supplementary material for: The impact of subacromial impingement syndrome on muscle activity patterns of the shoulder complex: a systematic review of electromyographic studies
Source: BMC Musculoskelet Disord. 2010 Mar 9;11:45. doi: 10.1186/1471-2474-11-45 (PMC2846868; doi:10.1186/1471-2474-11-45)
Supplement: Additional file 1 — Search strategy. Description of MESH terms and text words used in the literature search [file 1471-2474-11-45-S1.DOC]

Additional file 1. Search Strategy

**MEDLINE**

Region

1.*MESH terms:* exp Shoulder Joint/ or exp Shoulder/ or exp Shoulder Pain/

2. *Text words:* (shoulder$ or gleno-humer$ or scapulo-humer$ or humero-scapul$).mp. [mp=title, original title, abstract, name of substance word, subject heading word]

3. 1 or 2

Condition

4. *MESH terms*: exp Rotator cuff/ or exp Subacromial impingement syndrome

5. Text *words:* (rotator cuff$ or subacromial impingement$) mp. [mp=title, original title, abstract, name of substance word, subject heading word]

6. 4 or 5

Measurement

7. *MESH terms:* exp electromyography

8. Text *words:* (electromyograph$) mp. [mp=title, original title, abstract, name of substance word, subject heading word]

9. 7 or 8

Combination of terms

3 and 6 and 9

Similar searches based on the above format took place in Embase, Amed and Cinahl
