# Supplementary material for: The impact of subacromial impingement syndrome on muscle activity patterns of the shoulder complex: a systematic review of electromyographic studies
Source: BMC Musculoskelet Disord. 2010 Mar 9;11:45. doi: 10.1186/1471-2474-11-45 (PMC2846868; doi:10.1186/1471-2474-11-45)
Supplement: Additional file 2 — Population characteristics and procedural details. Subject and control characteristics, details of task and EMG collection [file 1471-2474-11-45-S2.DOC]

Additional File 2: Population characteristics and procedural details.

Note: Cools et al 2003 and 2007, same participants used for both outcomes/papers.

Moraes et al 2008 and Morais Faria et al 2008, same participants used for both outcomes/papers

| **Paper and country of origin** | **Subject details**  **Shoulders:** | **Asymptomatic control details** | **Task (test procedures and functional movement)** | **Electrode type; sampling rate; signal processing; normalisation** | **EMG variables analysed** | **Shoulder Pain present/absent during testing** |
| --- | --- | --- | --- | --- | --- | --- |
|  | **No starting /completing the study the study**  **M/F: Male/Female:**  **Age (years): ±SD**  **Participants characteristics**  **Symptom duration** | |  |  |  |  |
|  |  |  |  |  |  |  |
| Bandholm et al, 2006 Denmark | **No. start/completion** 9  **M/F** unclear  **Age** 28.2 ± 5.3 (21-38)  **Participant characteristics** Unilateral SAI. Recurrent Shoulder pain >2 months.  Minimum of two weekly sessions in upper limb strength training or physical activity in last 3 months.  **Examination** +ve painful arch, +ve Hawkins.  No history of other Sh/neck disorders (verified by clinical examination and US scan).  **Symptom duration** 27.6 ± 27.7 months (5-72 months) | **No. start /completion** 9  **M/F** unclear  **Age** 27.7 ± 4.2 (22-37)  **Participant characteristics**  Matched with cases for age, height, body weight and involvement in upper limb sports and strength training. | In Kin-Com, elbow flexion 90°. Isometric MVC shoulder scaption 90° in int/ ext/zero rotn at 20, 27.5 and 35%MVC. Isokinetic Abduction recorded concentrically 40-45° and 95-100°, ecc at 110-95° and 55-40°  . | sEMG of anterior and middle deltoid, upper and lower trapezius, latissimus dorsi, serratus anterior. iEMG of infraspinatus and supraspinatus. SR 1000 Hz. Processed by RMS, window size NS. Normalised to levels during MVICs (abduction, adduction, internal & external rotation) with shoulder at 45° abduction | Average normalised EMG amplitude during:  i) Isometric contractions (9 sec)  ii) Conc and ecc isokinetic contractions during 40-55 and 95-110 degrees abd in scapular plane.. | Present. Pain measured on 10 point 100mm visual analogue scale. Subjects had more pain at rest and during tests than controls (p<0.05). |
|  |  |  |  |  |  |  |
| Brox et al (1997) Norway | **No. start/completion** 10  **M/F**  7/3  **Age** 38.5 (25-51)  **Participant characteristics** Unilateral rotator cuff tendinosis. 6/10 subjects receiving workers compensation.  **Examination** painful arch, pain on isometric abduction and/or rotation, +ve impingement sign.  Pain relief on SA injection of lidocaine.  Excluded subjects with rotator cuff rupture, arthritis, instability, neck or widespread joint involvement and difficulty relaxing.  **Symptom duration** minimum 3 months (median range 1-2 years). | **No. start/completion**: 9  **M/F** 4/5  **Age** 37 (27-62)  **Participant characteristics**  Academic professionals with no history of shoulder pain. | MVIC and subMVIC at 45° isometric scaption, in 90° elbow F. | sEMG of upper trapezius, middle deltoid, infraspinatus, iEMG of supraspinatus. SR 1000 Hz.. Processed by FWR and integrated over consecutive 0.4 sec windows. Normalised to levels during 3 sec MVIC (abduction) with shoulder at 45° abduction, | Average EMG amplitude (normalised and non-normalised) during:  i) sustained 25% subMVIC until exhaustion  ii) MVICs (3 sec) at 30 sec, 3 min and 20 min post-exhaustion | Present. Full details for each test. Increase from 10%±3% of VAS at rest to 28%±4% after 4^th^ MVC and 48%±6% at exhaustion. |
|  |  |  |  |  |  |  |
| Clisby et al (2008) Australia | **No start/completion** 15/14 (18 shoulders as 4 bilateral)  **M/F** 5/9  **Age** 51.1±11.1  Height 168.3±10.8  Weight 78.6±24.2  **Participant characteristics/examination** SAI based on pain anterior/lateral sh. with active elevation, resisted abduction 90 degrees, +ve Hawkins.  No cervical or shoulder pathology (including rotator cuff tear) confirmed on x-ray  **Symptom duration** unclear | **No start/completion**: 18/18 (18 shoulders),  **M/F** N/D  **Age** 42.2±7.6  Height: N/D “similar”  Weight 69.9± 7.6  **Participant characteristics** “Asymptomatic”. No further details. | Resisted isometric external rotation at 10%, 40% and 70% MVC with and without shoulder adduction. | sEMG of infraspinatus, posterior deltoid, and middle deltoid.  SR NS. Processed by RMS, window size NS. Normalised to levels during 5 sec external rotation MVIC | Average normalised EMG amplitude during  the middle 5 seconds of the different contraction levels. | Not assessed |
|  |  |  |  |  |  |  |
| Cools et al (2003)  Belgium  (Onset times) | **No. start/completion** 39  **M/F** 26/13  **Age** 25.9±5.9 (16-35)  Height 178±8  Weight 73.6±9.8  **Participant characteristics** Unilateral SAI. Participation in overhand upper limb sports. No trauma or neck involvement.  **Examination** +ve for following 2/5 tests: neer, hawkins, Jobe, pain rather than apprehension on apprehension and relief on relocation.  **Symptom duration** 12.4±11.3 months | **No. start/completion** 30  **M/F** 19/11  **Age** 22.5±4.3 (18-36)  Height 178±9  Weight 68.6±9.3  **Participant characteristics** Overhead upper limb sports, with no history of shoulder injury. | Seated in Biodex isokinetic dynamometer. Action: to resist an unexpected drop (release of lever arm from locked position) when sh in 90° abdn. | sEMG of upper, middle and lower trapezius, middle deltoid. SR 1000 Hz. Processed by FWR & 6 Hz LPF. Normalisation N/A. | EMG onset times determined as point at which signal exceeded 10% of MVIC amplitude. Latencies (muscle response times in test) calculated. | Not documented |
|  |  |  |  |  |  |  |
| Cools et al (2007) Belgium  (%MVC_EMG_) | As above | As above | Seated on Biodex, isokinetic dynamometer Sitting, trunk fixation. i) Isokinetic abduction/ abduction in frontal plane 120°/sec ii) external rotation in 45°scaption (30° anterior to coronal plane) 60°/sec. | sEMG upper, middle and lower trapezius. SR 1000Hz.  Processed by FWR & 6 Hz LPF. Normalised to peak 50 msec average of 5 sec manual MVICs | Average normalised EMG amplitude of the 5 repetitions of each activity. | As above |
|  |  |  |  |  |  |  |
| Finley et al (2005) USA | **No. start/completion** 10  **M/F** 10/0  **Age** 44.1±3.0*  Height: 170±2*  Weight: 77.6±4.9*  **Participant characteristics** Wheelchair user (majority spinal cord injury). SAI -  **Examination**. Fulfilled minimum of 4 of 6 criteria – +ve neer, Hawkins, arc, pain anterior/lateral shoulder, tenderness on palpation, pain on resisted abduction.  **Symptom duration** unclear | **No. start/completion** 13  **M/F** 13/0  **Age** 41.7±2.6*  Height 180±3*  Weight 75.8±3.5*  **Participant characteristics** Wheelchair user (majority spinal cord injury). No History of shoulder pain and negative impingement tests | Wheelchair transfers from chair to bed from dominant and non-dominant sides. | sEMG of biceps brachii, anterior deltoid, upper trapezius, serratus anterior, lower trapezius. SR 1000Hz. Processed by FWR & 6 Hz LPF. Normalised to peak values during manual MVICs for each muscle. | Normalised peak EMG amplitude during phases  of transfer (each 30 degree of humeral elevation). | Not documented |
|  |  |  |  |  |  |  |
| Ludewig and Cook (2000) USA | **No. start/completion** 26 /25  **M/F** 26/0  **Age** 39.7± 12.0  Height 181±6.0  Weight: 90.9±14.0  **Participant characteristics** SAI. Construction workers with dominant upper extremity work > 1year in sheet metal and carpentry trades. Pain localised to proximal anterior/lateral shoulder. No neck or past shoulder injury  **Examination**. Abduction≥130 degrees, +ve impingement sign and painful arc.  **Symptom duration** 5.5±3.2 years (0.6-10) | **No. start/completion** 26/25  **M/F** 26/0  **Age** 39.9± 13.3  Height: 180±8.0  Weight: 85.7±12.7  **Participant characteristics** Construction workers with dominant upper extremity work in sheet metal and carpentry trades≥1yr. No history of shoulder injury or pain. Abduction ≥150 degrees | Standing, unilateral scaption (40 degrees ant to coronal) with and without a 5 or 10 lb (2.3/4.6 kg) load. One cycle every 4 seconds guided by metronome and flat surface | sEMG of upper, lower trapezius, lower serratus anterior.  SR 300 Hz. Processed by RMS, window size 25 msec. Normalised to peak 0.5 sec average during 3 sec manual MVICs. with arm at 75° scaption | Average normalised EMG amplitude for each of the 3 phases of humeral elevation (31-60,61-90, 91-120 degrees), taken from the middle 3 of 5 trials | Not documented |
|  |  |  |  |  |  |  |
| Moraes et al (2008) Brazil.  (Onset times) | **No. start/completion** 10  **M/F** (unclear 4/6 or 6/4)  **Age** 28.6±5.9 (20-38)  Height: 165±9  Weight: 60.3±11.8  **Participant characteristics** SAI grade I/II diagnosed by an Orthopaedic Surgeon. 8/10 sedentary lifestyle. Excluded if previous physiotherapy, general joint changes or pain affecting ability to perform the tests.  **Symptom duration** 2.8 (±1.6) months (range 1-6 months) | **No start/completion** 10  **M/F** 6/4  **Age** 29.0±5.4 (21-36) Height: 166±6  Weight: 58.2±11.1  **Participant characteristics** No history of shoulder disorder. | Standing, bilateral scaption, (30° ant to coronal plane) position guided flat surface. Performed at comfortable speed on verbal command | sEMG of serratus anterior, upper, middle, & lower trapezius, (bilateral). SR NS. Processed by FWR & LPF (cut-off NS). | EMG onset times (relative to verbal command) determined as the point at which signal exceeded mean baseline by 2 SD for 50ms. | Not documented although subjects were excluded if pain present to an extent which could affect their ability to perform the tests. |
|  |  |  |  |  |  |  |
| Morais Faria et al (2008)  Brazil  (%MVC_EMG_) | As above | As above | Standing, lowering the arms from full bilateral scaption (30 degrees anterior to the frontal plane). Performed at “comfortable speed” guided flat surface | sEMG of serratus anterior, upper, middle, & lower trapeziusSR 1000 Hz. Processed by FWR & LPF (cut-off NS Normalised to peak 2 sec average during 6 sec MVICs | Average normalised EMG amplitude from 3 repetitions of arm lowering, split into 6 phases:.. 1)full elevation to 150 degrees, 2)150 to 120 degrees 3)120 to 90 degrees, 4)90 to 60 degrees, 5)60 to 30 degrees and 6)30 to 0 degrees. Coactivation ratios also calculated. | As above |
|  |  |  |  |  |  |  |
| Reddy et al (2000) USA | **No start/completion** 15  **M/F** 12/3  **Age** 53.5 (40-66)  Height N/D  Weight: N/D  **Participant characteristics** SAI. On waiting list for shoulder arthroscopy and subacromial decompression. Minimum of 3 months of unsuccessful Physiotherapy. Diagnosis confirmed at surgery. Subjects with instability or full thickness tears of rotator cuff excluded.  **Symptom duration** Min 3 months | **No start/completion** 15 (abstract – not documented in text)  **M/F** N/D  **Age**: N/D  Height: N/D  Weight: N/D  **Participant characteristics** N/D | Scaption from 0 to 120 degrees elevation, elbow straight, whilst holding a weight which is 25% of their NMW. 100 degrees per second guided by metronome and flat surface (second beat of metronome full elevation, 3^rd^ beat return to start) | iEMG of middle deltoid, supraspinatus, infraspinatus, subscapularis and teres minor. SR 2500Hz. Processing not clearly stated, probably integration. Normalised to peak 0.5 sec average during 5 sec manual MVICs | Average normalised EMG amplitude for each phase of scaption (30-60, 60-90 and 90-120 degrees) | Not documented |
|  |  |  |  |  |  |  |
| Wadsworth and Bullock-Saxton (1997) | **No. start/completion** 9  **M/F** N/D  **Age** 23.2  Height: N/D  Weight: N/D  **Participant characteristics** Competitive freestyle swimmers swimming 30.2km/week on average over past 8.2 years. Unilateral shoulder pain. No history of contra-lateral shoulder pain.  **Examination** positive “impingement sign”, negative apprehension sign.  **Symptom duration** at least 3 weeks (mean 6.7 months) | **No. start/completion** 9  **M/F** N/A  **Age** 19.3  Height: N/D  Weight: N/D  **Participant characteristics** Competitive freestyle swimmers with no shoulder injury for the past 3 years. | Standing, Scaption to 160° (30 degrees anterior to the frontal plane). Guided by vertical guiders and metronome to achieve 40°arc of movement per sec. | sEMG of upper trapezius, lower trapezius, serratus anterior (assessed bilaterally). SR 2500 Hz. Processed by FWR and smoothing, details NS. Normalisation N/A | EMG onset times (relative to movement) determined as point at which signal exceeded 5% of maximum amplitude (and visual determination for serratus anterior). | Not documented |

Abbreviations: EMG - electromyography; F – Female; FWR – full wave rectification; iEMG intramuscular electromyography; LPF – low pass filter; M – Male; ms – milliseconds; MVC – maximal voluntary contraction; MVIC – maximal voluntary isometric contraction; ND: Not documented; No. – Number; RMS – root mean square; SD- standard deviation; sec – seconds; sEMG – surface electromyography; SR – sampling rate; VAS – visual analogue scale
