# Supplementary material for: The impact of subacromial impingement syndrome on muscle activity patterns of the shoulder complex: a systematic review of electromyographic studies
Source: BMC Musculoskelet Disord. 2010 Mar 9;11:45. doi: 10.1186/1471-2474-11-45 (PMC2846868; doi:10.1186/1471-2474-11-45)
Supplement: Additional file 3 — Mean differences (Mean diff.) 95% confidence intervals (95%CI) and statistical significance of differences in Supraspinatus %MVC(EMG) activity between subjects with (Subjects) and without (Controls) SIS. [file 1471-2474-11-45-S3.DOC]

Additional File 3: Mean differences (Mean diff.) 95% confidence intervals (95%CI) and statistical significance of differences in Supraspinatus %MVC_(EMG)_ activity between subjects with (Subjects) and without (Controls) SIS.

| **Author** | **Task** | **Torque as %MVC _(EMG)_** | **Subjects** | **Controls** | **Mean diff.** | **95%CI** | **Stat. sig.** |
| --- | --- | --- | --- | --- | --- | --- | --- |
|  |  | |  |  |  |  |  |
|  | Concentric Scaption | |  |  |  |  |  |
| Bandholm | <60° | 20%MVC | 20.2±4.3 | 18.1±4.3 | 2.09 | -1.8, 6.0 | 0.30 |
| Reddy |  | 25% MVC | 62 | 79 |  |  | >0.05 |
| Bandholm |  | 27.5% MVC | 25.5±3.9 | 24.9±6.0 | 0.65 | -4.0, 5.3 | 0.78 |
| Bandholm |  | 35%MVC | 31.6±5.5 | 31.3±9.7 | 0.34 | -7.0, 7.6 | 0.93 |
| Reddy | 60°-90° | 25% MVC | 79 | 87 |  |  | >0.05 |
| Bandholm | >90° | 20%MVC | 26.3±8.9 | 23.9±5.8 | 3.40 | -3.5, 10.3 | 0.34 |
| Reddy |  | 25% MVC | 82 | 85 |  |  | >0.05 |
| Bandholm |  | 27.5% MVC | 34.2±12.0 | 31.2±8.1 | 3.04 | -6.4, 12.5 | 0.53 |
| Bandholm |  | 35%MVC | 41.6±13.4 | 42.8±10.7 | -1.15 | -12.3, 10.1 | 0.84 |
|  |  |  |  |  |  |  |  |
|  | Eccentric Scaption | |  |  |  |  |  |
| Bandholm | <60° | 20%MVC | 18.6±2.6 | 15.9±5.3 | 2.63 | -1.5, 6.7 | 0.21 |
| Bandholm |  | 27.5% MVC | 25.3±2.6 | 23.7±8 | 1.64 | -3.9, 7.1 | 0.56 |
| Bandholm |  | 35% MVC | 32.2±3.7 | 30.6±12.1 | 1.53 | -6.7, 9.8 | 0.72 |
| Bandholm | >90° | 20%MVC | 20.3±3.3 | 16.8±4.2 | 3.46 | -0.1, 7.0 | 0.05 |
| Bandholm |  | 27.5% MVC | 26.7±5.2 | 34.3±5.6 | 2.41 | -2.6, 7.4 | 0.34 |
| Bandholm |  | 35% MVC | 35.7±8.2 | 34.3±8.1 | 1.32 | -6.2, 8.8 | 0.73 |
|  |  |  |  |  |  |  |  |
|  | Isometric Scaption | |  |  |  |  |  |
| Bandholm | 90° | 20%MVC | 21.0±3.6 | 19.5±6.2 | 1.55 | -3.2, 6.3 | 0.52 |
| Bandholm |  | 27.5% MVC | 31.9±6.4 | 28.0±9.1 | 3.98 | -3.3, 11.3 | 0.28 |
| Bandholm |  | 35% MVC | 39.9±6.5 | 37.2±7.0 | 2.68 | -3.6, 8.9 | 0.40 |
| Brox | 45° | Mean 25% MVC | 53±57.5 | 46±24.4 | 7 | -32.0, 46.0 | 0.73 |
| Brox |  | 25% MVC @ exhaustion | 64±52.5 | 49±22.7 | 15 | -20.8, 50.8 | 0.73 |
| Brox |  | 25% MVC after 10 mins. recovery | 45±26.2 | 29±17.4 | 16 | -3.8, 35.8 | 0.11 |
|  |  |  |  |  |  |  |  |
