# Supplementary material for: The impact of subacromial impingement syndrome on muscle activity patterns of the shoulder complex: a systematic review of electromyographic studies
Source: BMC Musculoskelet Disord. 2010 Mar 9;11:45. doi: 10.1186/1471-2474-11-45 (PMC2846868; doi:10.1186/1471-2474-11-45)
Supplement: Additional file 4 — Mean differences (Mean diff.) 95% confidence intervals (95%CI) and statistical significance of differences in Infraspinatus %MVC(EMG) activity between subjects with (Subjects) and without (Controls) SIS. [file 1471-2474-11-45-S4.DOC]

Additional file 4: Mean differences (Mean diff.) 95% confidence intervals (95%CI) and statistical significance of differences in Infraspinatus %MVC_(EMG)_ activity between subjects with (Subjects) and without (Controls) SIS.

| **Author** | **Task** | **Torque as %MVC _(EMG)_** | **Subjects** | **Controls** | **Mean diff.** | **95%CI** | **Stat.sig** |
| --- | --- | --- | --- | --- | --- | --- | --- |
|  |  |  |  |  |  |  |  |
|  | Concentric scaption | |  |  |  |  |  |
| Bandholm | <60° | 20%MVC | 13.5±5.3 | 14.2±2.8 | -0.77 | -4.7, 3.1 | 0.70 |
| Reddy |  | 25% MVC | 32 | 60 |  |  | *< 0.05 |
| Bandholm |  | 27.5% MVC | 15.4±7.3 | 16.9±2.8 | -1.48 | -6.6, 3.6 | 0.57 |
| Bandholm |  | 35%MVC | 20.6±10.8 | 21.0±4.1 | -0.41 | -7.9, 7.12 | 0.92 |
| Reddy | 60°-90° | 25% MVC | 43 | 60 |  |  | *<0.05 |
| Bandholm | >90° | 20%MVC | 15.6±7.5 | 14.7±3.8 | 0.94 | -4.5, 6.4 | 0.74 |
| Reddy |  | 25% MVC | 36 | 49 |  |  | >0.05 |
| Bandholm |  | 27.5% MVC | 18.5±8.3 | 19.6±6.1 | -1.11 | -7.8, 5.6 | 0.75 |
| Bandholm |  | 35%MVC | 25.2±9.7 | 24.6±6.8 | 0.63 | -7.1, 8.4 | 0.87 |
|  |  |  |  |  |  |  |  |
|  | Eccentric scaption | |  |  |  |  |  |
| Bandholm | <60° | 20%MVC | 13.0±4.8 | 11.7±2.3 | 1.28 | -2.2, 4.8 | 0.47 |
| Bandholm |  | 27.5% MVC | 16.7±7.2 | 15.1±1.9 | 1.61 | -3.3, 6.5 | 0.52 |
| Bandholm |  | 35% MVC | 23.6±8.6 | 18.6±2.3 | 4.96 | -0.9, 10.8 | 0.10 |
| Bandholm | >90° | 20%MVC | 14.2±5.3 | 13.3±3.5 | 0.91 | -3.2, 5.1 | 0.67 |
| Bandholm |  | 27.5% MVC | 18.7±9.2 | 17.4±5.2 | 1.39 | -5.5, 8.3 | 0.69 |
| Bandholm |  | 35% MVC | 26.3±10.9 | 21.4±6.7 | 5.3 | -3.1, 13.6 | 0.22 |
|  |  |  |  |  |  |  |  |
|  | Isometric scaption | |  |  |  |  |  |
| Bandholm | 90° | 20%MVC | 10.6±5.0 | 11.0±2.8 | -0.33 | -4.1, 3.4 | 0.86 |
| Bandholm |  | 27.5% MVC | 15.2±9.1 | 13.7±3.8 | 1.50 | -4.9, 7.9 | 0.64 |
| Bandholm |  | 35% MVC | 21.0±10.5 | 18.9±4.6 | 2.12 | -5.41, 9.7 | 0.58 |
| Brox | 45° | Mean 25% MVC | 35.0±38.8 | 23.0±13.9 | 12.00 | -13.7, 37.7 | 0.36 |
| Brox |  | 25%MVC @ exhaustion | 54.0±26.2 | 48±26.2 | 6.00 | -17.6, 29.6 | 0.62 |
| Brox |  | 25% MVC after 10 mins. recovery | 48.0±21.8 | 23.0±13.1 | 17.00 | 1.0, 33.0 | *0.04 |
|  |  |  |  |  |  |  |  |
|  | Isometric external rotation | |  |  |  |  |  |
| Clisby | 10% MVC | | 35.0±7.9 | 31.0±9.1 | 4.00 | -1.9, 9.0 | 0.18 |
| Clisby | 10% MVC + adduction | | 32.0±7.9 | 30.5±7.9 | 1.50 | -4.1, 7.1 | 0.60 |
| Clisby | 40% MVC | | 39.0±11.3 | 37.0±11.3 | 2.00 | -5.9, 9.92 | 0.62 |
| Clisby | 40% MVC + adduction | | 39.0±9.1 | 37.0±9.1 | 2.00 | -4.3, 8.3 | 0.54 |
| Clisby | 70% MVC | | 33.0±10.2 | 33.5±11.3 | -0.50 | -8.0, 7.0 | 0.90 |
| Clisby | 70% MVC + adduction | | 34.0±9.1 | 35.0±11.3 | -1.00 | -8.7, 6.1 | 0.78 |
