# Supplementary material for: The impact of subacromial impingement syndrome on muscle activity patterns of the shoulder complex: a systematic review of electromyographic studies
Source: BMC Musculoskelet Disord. 2010 Mar 9;11:45. doi: 10.1186/1471-2474-11-45 (PMC2846868; doi:10.1186/1471-2474-11-45)
Supplement: Additional file 5 — Mean differences and 95% confidence intervals (95%CI) for differences in Middle Trapezius %MVC(EMG) activity between subjects with (Subjects) and without (Controls) SIS. [file 1471-2474-11-45-S5.DOC]

Additional file 5: Mean differences and 95% confidence intervals (95%CI) for differences in Middle Trapezius %MVC_(EMG)_ activity between subjects with (Subjects) and without (Controls) SIS.
