# Supplementary material for: The impact of subacromial impingement syndrome on muscle activity patterns of the shoulder complex: a systematic review of electromyographic studies
Source: BMC Musculoskelet Disord. 2010 Mar 9;11:45. doi: 10.1186/1471-2474-11-45 (PMC2846868; doi:10.1186/1471-2474-11-45)
Supplement: Additional file 6 — Mean differences and 95% Confidence Intervals of differences in Lower Trapezius EMG activity between subjects with (Subjects) and without (Controls) SIS. [file 1471-2474-11-45-S6.DOC]

Additional file 6: Mean differences and 95% Confidence Intervals of differences in Lower Trapezius EMG activity between subjects with (Subjects) and without (Controls) SIS.

**Concentric Scaption**

**Eccentric Scaption**

**Wheelchair Transfers**

**a) Towards the affected Shoulder**

**b) Away from the affected shoulder**

**Isokinetic external rotation and Isometric abduction**
